# Monitoring of PCDD/Fs and PCBs in European Eels (*Anguilla anguilla*) from Lake Garda: A Persistent Environmental Concern

**DOI:** 10.3390/toxics13080690

**Published:** 2025-08-19

**Authors:** Federica Gallocchio, Marzia Mancin, Aurora Boscolo Anzoletti, Roberto Angeletti, Giancarlo Biancotto, Giorgio Fedrizzi, Mara Gasparini, Barbara Angelone, Silvana Bontacchio, Sabrina Di Millo, Francesca Cito, Gianfranco Diletti, Giuseppe Arcangeli

**Affiliations:** 1 Istituto Zooprofilattico Sperimentale delle Venezie, Viale dell’Università, 10, 35020 Legnaro, Italy; aboscolo@izsvenezie.it (A.B.A.); rangeletti@hotmail.it (R.A.); gbiancotto@izsvenezie.it (G.B.); garcangeli@izsvenezie.it (G.A.); 2 Istituto Zooprofilattico Sperimentale della Lombardia e Dell’Emilia Romagna, Via Antonio Bianchi, 7/9, 25124 Brescia, Italy; giorgio.fedrizzi@izsler.it (G.F.); mara.gasparini@izsler.it (M.G.); barbara.angelone@izsler.it (B.A.); sabrina.dimillo@izsler.it (S.D.M.); 3 Istituto Zooprofilattico Sperimentale dell’Abruzzo e del Molise Giuseppe Caporale, Via Campo Boario, 1, 64100 Teramo, Italy; f.cito@izs.it (F.C.); g.diletti@izs.it (G.D.)

**Keywords:** dioxin, PCBs, chemical pollutants, eels, Lake Garda

## Abstract

This study investigates the concentrations and patterns of dioxins and dioxin-like PCBs (TEQ Diox+PCB-DL) and non-dioxin-like PCBs (PCB-NDL) in eels from Lake Garda, assessing their relationship with biometric and lipid parameters. TEQ Diox+PCB-DL levels ranged from 1.70 to 77.1 pg/g (median: 9.90 pg/g), while PCB-NDL levels spanned from 14.0 to 1620 ng/g (median: 65.5 ng/g). Significant, albeit low, correlations were found: length and weight were negatively correlated, and lipid content was positively correlated, with both contaminants. Multivariable regression confirmed length and lipid percentage as significant predictors, although the models explained a limited proportion of variance (R^2^: 0.23 and 0.17). Classification-based analyses showed that irregularly contaminated eels were shorter and had a higher lipid content. Multinomial logistic regression supported these findings, but showed limited predictive accuracy (AUC = 0.63). Notably, 28 out of 90 samples exceeded the EU regulatory limit for TEQ Diox+PCB-DL, and several surpassed the threshold for PCB-NDL, highlighting potential public health risks. Given the lipophilic nature and toxicity of these compounds, continued monitoring is warranted. The findings underscore the need for broader environmental assessments to better understand pollutant dynamics and support regulatory actions, including the extended ban on eel fishing in the region.

## 1. Introduction

The terms *dioxins* and *dioxin-like compounds* refer to a group of toxic and environmentally persistent organic pollutants (POPs), including polychlorinated dibenzo-*p*-dioxins (PCDDs), polychlorinated dibenzofurans (PCDFs), and specific congeners of polychlorinated biphenyls (PCBs) [1,2].

Dioxins and furans are unintentional byproducts of combustion processes (e.g., waste incineration, forest fires) and certain industrial activities, such as pulp and paper bleaching or the manufacturing of chlorinated pesticides [2]. In contrast, PCBs were intentionally synthesized and widely used in the past as dielectric and coolant fluids in electrical equipment, heat exchangers, and hydraulic systems [1,2,3].

Due to their toxicity and persistence in the environment, PCB production was banned by the U.S. Congress in 1979, and later by the Stockholm Convention on POPs in 2001 [4].

The toxicological behavior of PCBs is closely linked to their chemical structure: congeners with mono-ortho chlorine substitutions are classified as dioxin-like PCBs (dl-PCBs), whereas the remaining congeners are considered non-dioxin-like PCBs (ndl-PCBs) [3].

Dl-PCBs exert toxic effects primarily through activation of the aryl hydrocarbon receptor (AhR), similarly to PCDDs and PCDFs. These AhR agonists induce a wide range of toxic responses, including embryotoxicity, hepatotoxicity, immunotoxicity, teratogenicity, and carcinogenicity [5]. Ndl-PCBs follow different toxicological pathways independently of AhR, contributing to neurological, neuroendocrine, endocrine, immunological, and carcinogenic effects [6].

Although environmental concentrations of these contaminants have decreased since the late 1970s, concern remains due to their persistence and bioaccumulation in the food chain, particularly in animal fats [1,2,7]. Over the past decades, multiple studies have demonstrated the role of dioxins and dioxin-like compounds in the decline of the European eel (*Anguilla anguilla*), a species that is highly sensitive to these pollutants [5,8,9,10,11].

European eels are bottom-dwelling, long-living, carnivorous, and lipid-rich fish, making them especially prone to bioaccumulating lipophilic contaminants [3]. During their feeding phase as yellow eels, these pollutants accumulate in fat tissue, sometimes reaching levels that render them unsafe for human consumption [9]. Eels enter continental waters as glass eels and remain there for 6 to 20 years before undergoing metamorphosis into silver eels and beginning their oceanic migration—spanning approximately 5000–7500 km—to the Sargasso Sea, where reproduction occurs [12].

During this migration, silver eels rely exclusively on fat reserves for energy, as they are believed not to feed en route. The mobilization of fat increases internal concentrations of lipophilic pollutants, heightening the risk of toxic effects [9,11]. Moreover, fat reserves are essential for gonadal development. Dioxins and dl-PCBs have been associated with endocrine disruption, teratogenic and mutagenic effects, hepatic damage, and reproductive impairment in wildlife [3,8].

**Figure 1:**
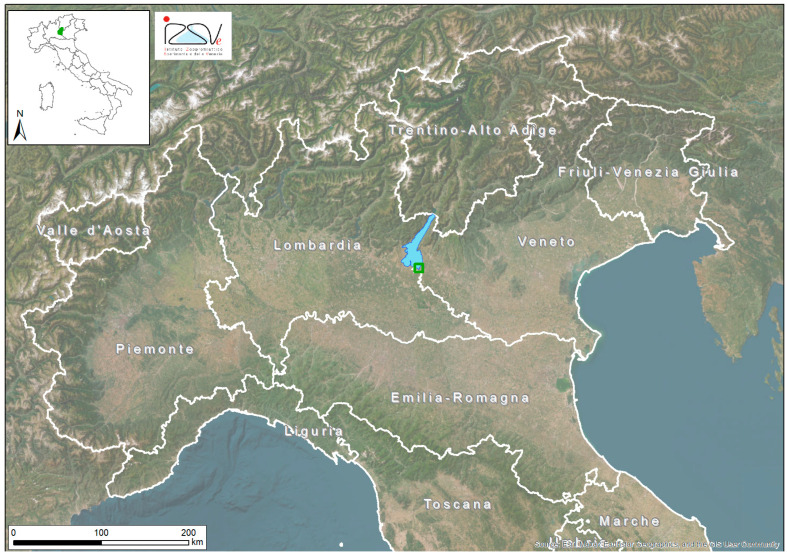
Map of sampling area (green square in the south part of Garda Lake).

Lake Garda, Italy’s largest lake (368 km^2^), is situated in one of the country’s most industrialized and densely populated regions, bordered by the Veneto and Lombardia regions and the Autonomous Province of Trento [13] (Figure 1).

The European eel was once a major catch in the lake, with peak landings of 70 tonnes/year during the 1980s, followed by a notable decline beginning in the early 2000s [14].

Fishing of eels in Lake Garda has been banned since 2011, following a monitoring program that detected levels of PCDD/Fs, dl-PCBs, and ndl-PCBs exceeding the regulatory thresholds set by Regulation (EC) No. 1881/2006, now superseded by Regulation (EU) 2023/915 [13,15]. A second monitoring campaign in 2016 confirmed continued contamination, and the ban remained in effect until 2023 [13].

This study aimed to carry out a third monitoring program to assess the current levels of PCDD/Fs, dl-PCBs, and ndl-PCBS in eels from Lake Garda. The objectives were as follows:

(1) Evaluate the contamination levels and profiles of PCDD/Fs, dl-PCBs, and ndl-PCBs in relation to regulatory maximum levels; 

(2) Assess the bioaccumulation of these compounds in relation to body weight, length, and fat content.

## 2. Materials and Methods

### 2.1. Chemical Reagents

Ethyl-acetate, toluene, and n-hexane were purchased from Carlo Erba Reagents (Milan, Italy); nonane was purchased from Promochem (LGC Standards, Teddington, UK); and dichloromethane was purchased from Romil LTD (Cambridge, UK). All solvents were picograde. Pre-packed multi-layer silica, alumina, and carbon columns were fabricated by FMS (Fluid Management System, Billerica, MA, USA). All solvents and reagents were tested to ensure the absence of contaminants at significant levels before they were for analysis.

All ^13^C_12_-labeled recovery, clean-up, and injection standard solutions were provided by CIL (Cambridge Isotope Laboratories, Andover, MA, USA). For PCDD/Fs, EDF-9999 Method 1613 calibration solutions (CS1-CS5), diluted five times, were used. For PCB calibration, an in-house curve was prepared using PCB MIX 1 and PCB MIX 41 (Dr. Ehrenstorfer, Augsburg, Germany), along with the 13C12-labeled solutions EC-4995, EC-4978, and EC-4979 (Cambridge Isotope Laboratories, Andover, MA, USA).

### 2.2. Sample Collection

The monitoring strategy was developed in agreement with the Lombardy Region, the Veneto Region, and the Autonomous Province of Trento. Eels were collected between April and June 2022 at the beginning of the Mincio River, the emissary river of Lake Garda, a place historically equipped for catching eels on their way down to the sea. In the entire lake, this site is the only one still prepared and authorized for this type of fishing.

For this purpose, the Veneto Region, through a specific decree (No. 287 of 29 March 2022), authorized a professional fisherman to collect eel specimens for the monitoring of PCDD/F and PCB contamination, as an exception to the fishing ban on the species established by the Decree of the President of the Veneto Region No. 120 of 17 August 2021, which implements the Ordinance of the Italian Ministry of Health dated 8 June 2021. The fishing activity was carried out by a by means of fish traps (Bertavello in the Italian language) consisting of a net with a single entrance and subsequent capture chambers, where the fish remain alive, but unable to return to the entry point.

In total, 90 adult eels (silver) were caught and divided in three different groups according to their size:

- A group of 30 eels with a length superior to 80 cm;
- A group of 30 eels with a length between 65 and 80 cm;
- A group of 30 eels with a length between 50 and 65 cm.

After sacrification, the eels were weighted, sized (all information is reported in Supplementary Materials Table S1), frozen, and transferred to a laboratory for analysis.

### 2.3. Chemical Analysis, Method Performance, and Quality Control

Chemical analyses were carried out at the Chemistry Laboratory of IZSLER (Istituto Zooprofilattico Sperimentale della Lombardia e dell’Emilia-Romagna).

The methods used for the determination of PCDD/Fs and PCBs were modifications of the methods US EPA 1613/B 1994 [16] and US EPA 1668/C 2010 [17]. The modifications were necessary to adapt the methods to the analyzed matrix and to assess the concentration of all congeners at background levels and levels of interest, according to current regulations. The laboratory that carried out the analyses is certified according to UNI CEI EN ISO/IEC 17025 and accredited for the quantitative determination of seventeen substituted PCDD/Fs, twelve PCB DL congeners (PCB 77-81-105-114-118-123-126-156-157-167-169-189), and six PCB NDLs (PCB indicators, 28-52-101-138-153-180) using high-resolution gas chromatography coupled with high-resolution mass spectrometry (HRGC-HRMS).

Before analysis, euthanasia was performed by immersion in tricaine methanesulfonate (MS-222) at a dose > 300 mg/L, in accordance with European legislation on animal experimentation. Subsequently, the eels were skinned and their entrails were removed; their heads, tails, and fishbones were discarded, and the residual fish muscles were homogenized and freeze-dried. During this process, the samples were subjected to several cycles of medium and high vacuum and low temperature to remove a large amount of water. After the freeze-drying process, the eel samples were homogenized again and a portion was used for the determination of lipid content using an accredited method.

Eel samples were weighed (2–4 g powder, depending on lipid content) and mixed with diatomaceous earth, then spiked with a mixture of fifteen ^13^C_12_-labeled PCDD/F and twelve ^13^C_12_-labeled PCB congeners. Extraction was performed with Accelerated Solvent Extraction (ASE) (Dionex, Sunnyvale, CA, USA); each sample was introduced into a specific cell and subjected to two extraction cycles, using toluene at 135 °C and 1500 psi. The solvent was filtered through anhydrous sodium sulfate and was removed from the matrix extract with a rotatory evaporator at 50 °C.

The extract fraction was resolubilized with 5 mL of hexane/dichloromethane solution (1:1 *v*/*v*), spiked with a standard clean-up solution containing three ^13^C_12_-labeled PCB congeners, and diluted with 20 mL of hexane.

Two purification steps were carried out. First, a column manually packed with Extrelut powder saturated with sulfuric acid (96% for analysis, Panreac ITW Companies Barcelona, Spain) was prepared to carbonize the fat component. The dilute extract was loaded into the packed column and eluted with 200 mL of hexane in a glass tube. The purification fraction was concentrated to 0.5 mL in a TurboVap evaporator (Zymark Corp., St. Hopkinton, MA, USA) at 35 °C and loaded into a Power-prep system (Fluid Management System, Waltham, MA, USA) equipped with silica, alumina, and carbon columns. This purification step was necessary to isolate analytes of interest from matrix interferences and to separate PCDD/F fractions from PCB ones. PCBs were eluted from the silica and alumina columns with n-hexane and a mixture of hexane/dichloromethane solution (90:10 *v*/*v*), while PCDD/Fs were eluted from the carbon column with toluene. Each final extract was evaporated to dryness in a TurboVap evaporator and in a vacuum concentrator (Genevac, Ipswich, UK). PCDD/F fractions were dissolved in ten microliters of ^13^C_12_ 1,2,3,4-TCDD and ^13^C_12_ 1,2,3,7,8,9-HxCDD injection solution, and PCB fractions in twenty microliters of ^13^C_12_ PCB 52-^13^C_12_ PCB 101-^13^C_12_ PCB 138-^13^C_12_ PCB 194 injection solution.

PCB and PCDD/F analysis was performed by two TRACE GC ULTRA gas chromatographers coupled to a high-resolution DFS magnetic scan system (Dual Focusing SystemThermoFisher, Bremen, Germany).

Chromatographic separation was performed on TR-Dioxin 5MS (60 m × 0.25 mm × 0.25 µm film thickness, ThermoFisher Scientific) for PCDD/Fs, and on TR-PCB 8MS (50 m × 0.25 mm × 0.25 µm film thickness, ThermoFisher Scientific) for PCBs.

The mass spectrometer, in selected ion monitoring (SIM) mode, operated at a resolution of over 10,000 during the acquisition time. For all congeners, two characteristic masses were monitored and quantification was performed using isotope dilution. TEQ values were calculated using 2005-WHO-Toxic Equivalency Factors [18]. Their presence in food is, in fact, regulated as the sum of equivalent toxicity expressed in pg/g toxic equivalents (TEQ).

Precision and accuracy were tested periodically as part of inter-laboratory studies; the laboratories involved have demonstrated successful participation in different international proficiency tests, such as InterCIND (LabService Analytica, Anzola dell’Emilia, BO, Italy); “Dioxin in Food” (Norwegian Institute of Public Health, Oslo, Norway); FAPAS (Fera Science LTD, York, UK); and EUPT tests organized by the European Reference Laboratory for Dioxins and PCBs in food and feed, obtaining acceptable z-scores. To prove the absence of interference with the congeners of interest and to prevent cross-contamination, a blank sample, i.e., without the presence of congeners, was analyzed in each batch of twelve samples. During each analytical batch, ongoing precision recovery (OPR) samples were tested, where the native PCDD/F and PCB standard (Cambridge Isotope Laboratories, Tewksbury, MA, USA) was added to the blank sample and processed under the same analytical conditions to assess the recovery rate.

According to US EPA methods, the stability of response factors for all congeners was checked using VER solutions for PCDD/Fs and PCBs, evaluating the acceptability of deviation from values calculated using calibration curves.

In each sample, the recovery of the labeled congeners was evaluated to monitor method performance according to the limits imposed by US EPA Methods. Repeatability was periodically tested, analyzing contaminated samples in duplicate and evaluating whether the standard deviation for each congener was comparable to that obtained during the validation study.

Further information about the analytical methods (LOQ and measurement units) is reported in the Supplementary Materials (Table S2).

The final results are expressed as wet weight.

### 2.4. Statistical Analysis

For convenience, from this point onward, PCDD/Fs will be referred to as Diox, and the sums will include TEQ as well.

Statistical analyses were performed on a dataset comprising 90 eels, from which the following variables were measured: length (in cm), weight (in kg), lipid percentage, and contaminant concentrations, expressed as TEQ Diox+PCB-DL and PCB-NDL. For both contaminant types, quantitative values and qualitative classifications in term of regular, irregular, and regular/uncertain were considered.

These classifications were established according to the maximum allowable levels defined by the former Regulation (EU) 1881/2006, now superseded by Regulation (EU) 915/2023. Specifically, eels were classified as “regular” if their Diox, Diox+PCB-DL, or PCB-NDL values were below 3.5 pg/g, 10 pg/g, or 300 ng/g wet weight, respectively; values exceeding these limits were classified as “irregular.” Measurements falling within the uncertainty interval were designated as “regular/uncertain.”

Additionally, an “overall” qualitative evaluation was obtained by combining the TEQ Diox+PCB-DL and PCB-NDL classifications.:“regular” if both were regular; “irregular” if either was irregular; and “regular/uncertain” if one was regular and the other was regular/uncertain.

#### 2.4.1. Quantitative Analysis of TEQ Diox+PCB-DL and PCB-NDL Compounds

Boxplots were used to synthesize data, providing the principal measures of central tendency and dispersion of the TEQ Diox+PCB-DL and PCB-NDL compounds. Scatter plots were used to show the relation between the two compounds and each of the variables of length, weight, and lipid percentage. Both the parametric Pearson and non-parametric Spearman indices were used to estimate the correlation between TEQ Diox+PCB-DL and PCB-NDL values and the physical variables. The interpretation of the correlations was based on the following rule of thumb [19]: a correlation between 0.90 and 1.00 (−0.90 to −1.00) was defined as a very high positive (negative) correlation; a correlation between 0.70 and 0.90 (−0.70 to −0.90) was defined as a high positive (negative) correlation; a correlation between 0.50 and 0.70 (−0.50 to −0.70) was defined as a moderate positive (negative) correlation; a correlation between 0.30 and 0.50 (−0.30 to −0.50) was defined as a low positive (negative) correlation; and, finally, a correlation between 0.00 and 0.30 (0.00 to −0.30) was defined as a negligible correlation.

Afterwards, a multivariable linear model was developed to assess the relationship of the quantitative levels of TEQ Diox+PCB-DL and PCB-NDL with two or more variables among length, weight, and lipid percentage. The influence of extreme values was assessed in model fitting to obtain more reliable estimation and enhanced robustness of the results. The goodness of fit of the nested model was evaluated using the adjusted R^2^.

#### 2.4.2. Qualitative Analysis on TEQ Diox+PCB-DL and PCB-NDL Compounds

Boxplots were used to synthesize data providing the principal measures of central tendency and dispersion of the variables length, weight, and lipid percentage according to the classifications (regular, irregular, and regular/uncertain) of TEQ Diox+PCB-DL, PCB-NDL, and “overall”.

The non-parametric Kruskal–Wallis test (for comparisons among more than two groups) and the Wilcoxon-Mann–Whitney test (for two groups) were performed to assess whether there were differences in the distribution of the variables length, weight, and lipid percentage according to the classifications of TEQ Diox+PCB-DL, PCB-NDL and “overall”. In the case of significance of the Kruskal–Wallis test, pairwise comparisons were performed using the Wilcoxon–Mann–Whitney non-parametric test with Holm correction.

Finally, a multinomial logistic regression model was applied to estimate the risk of an eel belonging to one class or another of TEQ Diox+PCB-DL, PCB-NDL, and “overall” as a function of its length, weight, and lipid percentage. To assess the goodness of fit of the model, the multi-class area under the roc curve (AUC) was evaluated. The multi-class AUC was calculated as the average AUC as defined by Hand and Till [20]:

$$AUC=\frac{2}{c(c-1)}\sum aucs,$$

where c is the number of classes and aucs represents the area under the ROC curve for each pairwise class comparison. A rough guide for classifying the accuracy of the models is as follows: AUC 0.9–1 = outstanding; 0.8–0.9 = excellent; 0.7–0.8 = acceptable; and 0.5–0.7 = poor [21].

A *p*-value < 0.05 was considered significant. R version 4.2.3 software [22] was used to perform the statistical analysis. The multinom function of the nnet package [23], and the auc and multiclass.roc functions of the pROC package [24] were applied.

## 3. Results

### 3.1. Chemical Analysis

None of the 90 eel samples exceeded the Maximum Residue Limit (MRL) of 3.5 pg/g fresh weight established by Regulation (EC) No. 1881/2006 for the sum of dioxins (PCDD/F). However, 28 out of 90 eel samples showed concentrations of polychlorinated dibenzo-p-dioxins and dibenzofurans (PCDD/F) and dioxin-like polychlorinated biphenyls (DL-PCBs) above the MRL of 10.0 pg/g fresh weight. Among these, 16 samples, while exceeding the MRL, would be considered compliant if taking measurement uncertainty into account.

Five samples exceeded the MRL of 300 ng/g fresh weight set for non-dioxin-like PCBs (NDL-PCBs); nonetheless, these samples were deemed non-compliant due to them exceeding the MRL for the combined total of PCDD/Fs and DL-PCBs.

The highest concentration of PCDD/Fs + DL-PCBs detected among the 90 eel samples was 77.1 pg WHO-TEQ/g, which corresponds to more than 7 times the MRL established by Regulation (EC) No. 1881/2006 for wild eel muscle. The highest concentration of NDL-PCBs detected was 1620 ng/g, exceeding by more than fivefold the MRL of 300 ng/g fresh weight for this class of organochlorine compounds.

Further detailed information is reported in the Supplementary Materials (Table S1).

### 3.2. Statistical Analysis

#### 3.2.1. Quantitative Analysis of the TEQ Diox+PCB-DL and PCB-NDL Compounds

**Figure 2:**
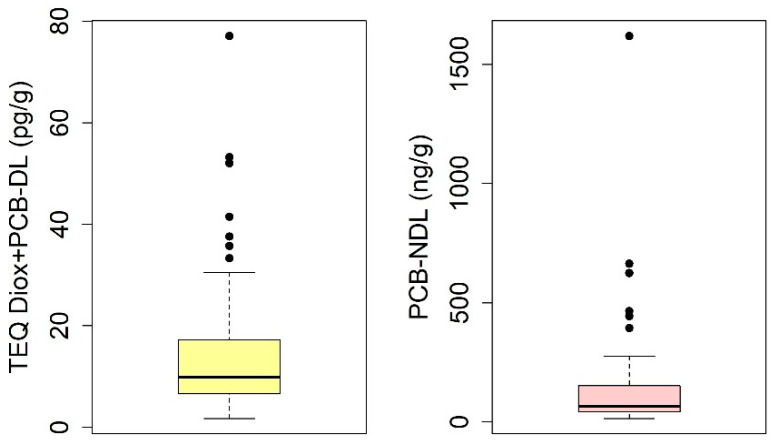
Boxplots of TEQ Diox+PCB-DL (**left panel**) and PCB-NDL (**right panel**) concentrations in the 90 analyzed eels. The horizontal line inside each box represents the median; the boxes cover the interquartile range (25th to 75th percentile). The whiskers extend to the 2.5th and 97.5th percentiles, while the dots represent extreme values (outliers).

The distribution of the TEQ Diox+PCB-DL and PCB-NDL values detected in the 90 analyzed eels is illustrated in Figure 2. The TEQ Diox+PCB-DL concentrations range from 1.70 to 77.10 pg/g, with a median of 9.90 pg/g, while the PCB-NDL concentrations vary between 14.0 and 1620.0 ng/g, with a median value of 65.5 ng/g (Supplementary Table S3). The boxplots summarize these data distributions (Figure 2).

**Figure 3:**
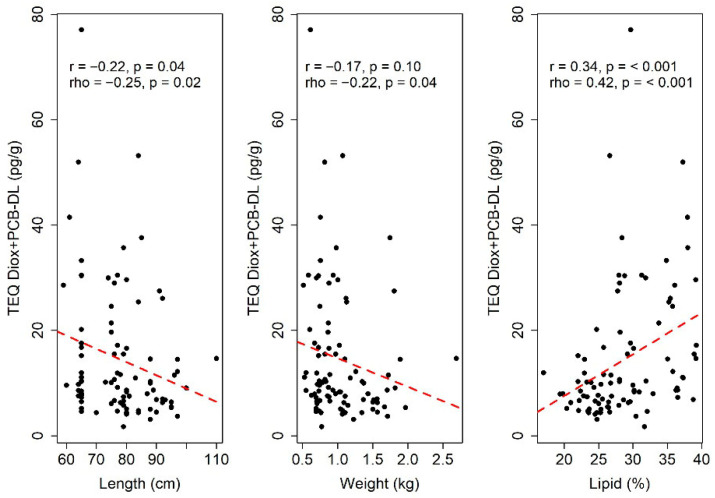
Correlation coefficients between TEQ Diox+PCB-DL and the variables of length, weight, and lipid percentage. r and rho denote Pearson’s and Spearman’s rank correlation coefficient values, respectively, and p represents the *p*-value. The red line describes a linear relationship.

**Figure 4:**
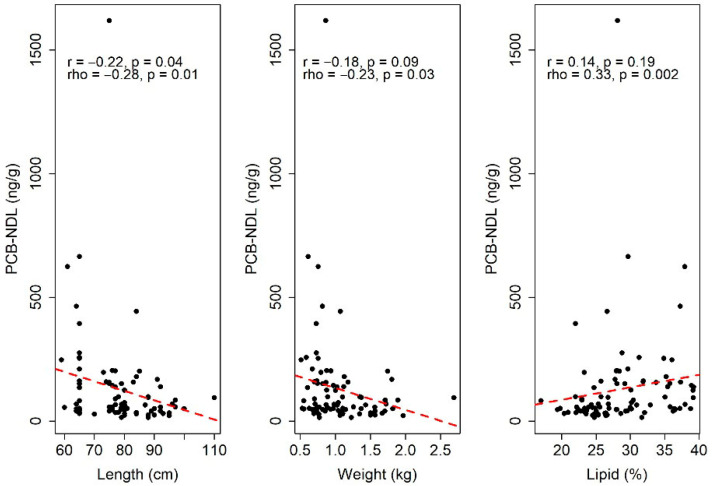
Correlation coefficients between PCB-NDL and the variables of length, weight, and lipid percentage. r and rho denote Pearson’s and Spearman’s rank correlation coefficient values, respectively, and p represents the *p*-value. The red line describes a linear relationship.

According to the Spearman correlation coefficient (rho), all variables are significantly correlated with TEQ Diox+PCB-DL (Figure 3). The correlation is negative for length and weight, and positive for lipid percentage. This indicates that an increase in length or weight is related to a decrease in TEQ Diox+PCB-DL levels, whereas an increase in lipid percentage corresponds to an increase in TEQ Diox+PCB-DL levels. However, based on rule of thumb, these correlations are considered negligible (for length and weight) and low (for lipid percentage). The same conclusions are valid also for PCB-NDL (Figure 4).

Based on the Pearson correlation coefficients (r), both length and lipid percentage are significantly correlated with TEQ Diox+PCB-DL. In addition, length is significantly linearly correlated with PCB-NDL.

Specifically, an increase in length is associated with a decrease in both TEQ Diox+PCB-DL and PCB-NDL values, whereas an increase in lipid percentage corresponds to an increase in TEQ Diox+PCB-DL levels.

Considering TEQ Diox+PCB-DL as the dependent variable in the multivariable linear model (weight, lipid percentage and length as independent variables), three observations were identified as outliers and were therefore removed from the analysis. A similar approach was applied for PCB-NDL, where only one observation was found to be an outlier. In both models, weight was not statistically significant, likely due to its high and significant correlation with length (Pearson’s r = 0.88, *p* < 0.001; Spearman’s rho = 0.90, *p* < 0.001). For this reason, a revised model excluding weight was developed. In the updated model, both remaining predictors, length and lipid percentage, were statistically significant, as reported in Table 1. Based on the estimated regression coefficients for TEQ Diox+PCB-DL, each 1 cm increase in length leads to a 0.21 pg/g decrease in TEQ Diox+PCB-DL, while each 1 percentage point increase in lipid content leads to a 0.76 pg/g increase in TEQ Diox+PCB-DL (Table 1).

For PCB-NDL, a 1 cm increase in length corresponds to a decrease of 3.85 ng/g in its concentration, whereas a 1 percentage point increase in lipids corresponds to an increase of 6.38 ng/g in its concentration (Table 1).

The adjusted R^2^ of the models is 0.23 and 0.17 for TEQ Diox+PCB-DL and PCB-NDL, respectively. This suggests that although the included predictors are statistically significant, they explain only 23% and 17% of the variability for TEQ Diox+PCB-DL and PCB-NDL, respectively, indicating that additional unaccounted factors may influence the outcomes.

#### 3.2.2. Qualitative Analysis of the Physical Variables According to the Classification of TEQ Diox+PCB-DL, PCB-NDL, and Overall

**Figure 5:**
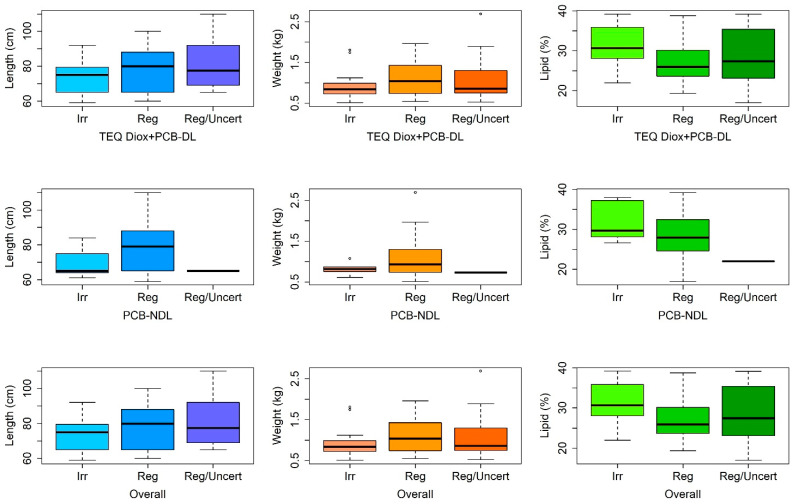
Boxplots of physical variables of the 90 analyzed eels according to the TEQ Diox+PCB-DL, PCB-NDL, and “overall” classifications. The horizontal line inside each box represents the median; the boxes cover the interquartile range (25th to 75th percentile). The whiskers extend to the 2.5th and 97.5th percentiles, while the dots represent extreme values (outliers).

Observations were reclassified as irregular, regular, or regular/uncertain based on their respective levels of TEQ Diox+PCB-DL and PCB-NDL. The “overall” classification, obtained by combining the individual classifications of TEQ Diox+PCB-DL and PCB-NDL, was determined solely by the classification of the first component. Consequently, it displays the same number of eels per category and the same descriptive statistics (Table 2 and Table S4 and Figure 5).

Boxplots of the variables of length, weight, and lipid percentage according to the classification of TEQ Diox+PCB-DL, PCB-NDL, and “overall” are reported in Figure 5.

The non-parametric Kruskal–Wallis test revealed a significant difference in median length and lipid percentage among eels classified as regular, irregular, and regular/uncertain based on their TEQ Diox+PCB-DL levels.

Specifically, the pairwise Wilcoxon–Mann–Whitney post hoc test with Holm correction indicated that regular eels had a significantly greater median length than irregular ones (*p* = 0.034), but a significantly lower median lipid percentage (*p* < 0.001). Although the Kruskal–Wallis test did not identify a significant overall difference in weight among the three groups, the pairwise Wilcoxon–Mann–Whitney test with Holm correction showed that regular eels were significantly heavier than irregular ones (*p* = 0.047).

Since the PCB-NDL compound classified only one eel as regular/uncertain, this category was excluded from the analysis in order to assess potential differences in the distribution of physical variables among the remaining categories. The non-parametric Wilcoxon–Mann–Whitney test was performed, and the results indicated no significant differences in the medians of length (*p* = 0.06), weight (*p* = 0.27), or lipid percentage (*p* = 0.17) between regular and irregular eels.

Based on the previous results, the multinomial logistic regression model was applied only to the TEQ Diox+PCB-DL compound. Consistent with the multivariable model constructed in the quantitative analysis, the optimal multinomial logistic model excluded the weight variable. Taking the regular category as the reference, the model indicated that both length and lipid percentage are significant predictors (*p* = 0.012 and *p* < 0.001, respectively). Specifically, holding all other variables constant, a 1 percentage point increase in lipid content makes eels 1.21 times more likely to fall into the irregular category rather than the regular one (i.e., 21% higher odds; Table 3). Similarly, a 1 cm increase in length makes eels 0.94 times as likely to be in the irregular category compared to the regular category (i.e., 6% lower odds; Table 3).

These findings are ecologically relevant, as they suggest that biological traits such as lipid content and body length influence contaminant accumulation patterns. Lipophilic pollutants like dioxins and PCBs are known to concentrate in fatty tissues; therefore, individuals with higher lipid reserves may accumulate greater contaminant loads, especially if exposure is chronic. Conversely, the association with shorter body length in irregular eels could reflect differences in age, growth dynamics, or habitat use, potentially linked to exposure pathways or feeding behavior. These traits may act as indirect indicators of contaminant exposure risk and could be useful in ecotoxicological monitoring frameworks.

The model’s predictive performance was limited, with a multi-class AUC of 0.63, which is generally considered poor. This result may be due to misclassification involving the regular/uncertain category, as well as the potential influence of unmeasured variables not included in the model (e.g., feeding habits, microhabitat characteristics, or local environmental concentrations of contaminants).

## 4. Discussion

This study provides an in-depth assessment of the levels and patterns of TEQ Diox+PCB-DL and PCB-NDL in eels, as well as their relationship with biometric and lipid parameters. The observed concentrations, ranging from 1.70 to 77.1 pg/g for TEQ Diox+PCB-DL and from 14.0 to 1620 ng/g for PCB-NDL, reveal high variability among samples, consistent with previous reports of spatial and biological heterogeneity in contaminant accumulation in aquatic species across Europe.

For example, a study conducted in the Loire estuary in France revealed average PCB concentrations in eel muscles ranging from 857 to 4358 ng/g lipid weight. Notably, 20% of the analyzed eels had toxic equivalent (TEQ) concentrations exceeding the maximum limits for lipid-rich species, indicating significant contamination levels [25].

A study conducted on eels from the Gironde estuary found that muscle tissue contamination was increased in silver eels compared to glass eels, with the most contaminated silver eel exhibiting PCB-NDL levels up to 3399 ng/g dry weight [26].

A study on eels from the lower Thames River reported detectable levels of NDL-PCBs in all sampled eels. The concentrations of NDL-PCBs (ICES7) ranged from 4.2 to 124 µg/kg fresh weight, with averages of 33 and 56 µg/kg for upstream and tidal eels, respectively [27].

In the Elbe River, concentrations of dioxins and dioxin-like PCBs in eel muscle tissue ranged from 0.48 to 22 pg WHO-TEQ/g wet weight for PCDD/Fs and from 8.5 to 59 pg WHO-TEQ/g wet weight for DL-PCBs. These findings highlight the significant contamination levels in eels from this region [28].

The results of this study align closely with previous research conducted on European eels (*Anguilla anguilla*) from Lake Garda by Chiesa et al. [13], particularly regarding the central tendency of contamination levels. Specifically, the median concentration of TEQ Diox+PCB-DL observed in the present study (9.90 pg/g wet weight) was comparable to that reported by Chiesa et al. (9.4 pg/g), while the PCB-NDL median levels (65.5 ng/g vs. 69.4 ng/g, respectively) also show consistency across studies. However, notable differences emerged in the upper range of concentrations. This study reported a maximum TEQ Diox+PCB-DL value of 77.1 pg/g and a PCB-NDL peak of 1620 ng/g, both considerably higher than the maxima reported by Chiesa et al. (50.8 pg/g and 735.3 ng/g, respectively), suggesting the presence of more heavily contaminated individuals or environmental hotspots in the sampled area.

Moreover, compared to the 2016 monitoring campaign, in which 18 out of 90 eel samples were found to be non-compliant, the 2022 survey showed an increase, with 28 out of 90 samples exceeding the regulatory limits [13].

It is well established that dioxins and related compounds preferentially accumulate in animal adipose tissue [1,2]. Over the past few decades, several studies have implicated these contaminants in the decline of the European eel (*Anguilla anguilla*), a species that is particularly vulnerable due to its high lipid content and associated bioaccumulation potential [5,8,9,10,11].

Correlation analyses indicated a statistically significant relationship between contaminant levels and biological variables. Specifically, TEQ Diox+PCB-DL and PCB-NDL levels were negatively correlated with eel length and weight, and positively correlated with lipid content. Although these correlations were statistically significant, their strength ranged from negligible to low according to standard interpretation thresholds. This suggests that while larger or heavier eels tend to exhibit lower pollutant concentrations, these physical traits alone are not strong predictors of contamination levels. In contrast, lipid content—due to the lipophilic nature of PCBs—showed a more meaningful relationship, particularly with TEQ Diox+PCB-DL.

Multivariable linear regression models further supported these findings. After excluding weight due to collinearity with length, both length and lipid percentage remained significant predictors. Each additional centimeter in length corresponded to a decrease in pollutant levels, while a higher lipid content led to increased contamination, reinforcing the bioaccumulative behavior of these compounds in lipid-rich tissues. However, the relatively limited adjusted R^2^ values (0.23 for TEQ Diox+PCB-DL and 0.17 for PCB-NDL) indicate that a considerable proportion of the variability remains unexplained, suggesting the potential role of additional environmental or biological factors that were not captured in this study.

The categorical classification of contamination levels (regular, irregular, regular/uncertain) enabled a more qualitative assessment of physical characteristics. Significant differences in length, weight, and lipid percentage were observed among the TEQ Diox+PCB-DL categories, consistent with the findings from the model. Notably, eels in the irregular group exhibited shorter lengths and higher lipid percentages than those classified as regular, reinforcing the inverse and direct relationships of these factors, respectively, with contamination levels.

For PCB-NDL, group-based comparisons revealed no significant differences across physical parameters, likely due to the very small number of irregular cases, whereas continuous models still demonstrated a significant influence of both length and lipid percentage.

The multinomial logistic regression applied to TEQ Diox+PCB-DL categories confirmed the significance of length and lipid percentage in predicting contamination status. Each 1% increase in lipid content was associated with 21% higher odds of belonging to the irregular group compared to the regular one, while each additional centimeter in length reduced the odds by 6%. However, the model’s predictive power was limited (AUC = 0.63), indicating challenges in classification accuracy—particularly for borderline cases in the regular/uncertain group—and underscoring the need to incorporate additional variables or refine the categorization thresholds.

Overall, the findings suggest that while biometric and lipid traits offer useful, albeit moderate, insights into PCB contamination levels in eels, a more comprehensive approach that incorporates environmental exposure factors, trophic dynamics, and possibly genomic data may be necessary to enhance predictive accuracy and fully elucidate patterns of contaminant accumulation.

Finally, evaluating contaminant concentrations in relation to current European Union food safety standards highlights potential health risks associated with the consumption of the eels analyzed in this study. According to Regulation (EU) 2023/915, the maximum allowable concentration for the sum of dioxins and dioxin-like PCBs (TEQ Diox+PCB-DL) in fish muscle is 10 pg WHO-TEQ/g wet weight, while for non-dioxin-like PCBs (PCB-NDL)—based on the sum of six indicator congeners—the limit is 300 ng/g wet weight. In this study, TEQ Diox+PCB-DL concentrations ranged from 1.70 to 77.1 pg/g, with a median of 9.90 pg/g, indicating that some eel samples exceeded the regulatory maximum. PCB-NDL concentrations ranged from 14.0 to 1620 ng/g, with a median of 65.5 ng/g. Although the median was within the legal limit, several samples exceeded the 300 ng/g threshold, with some reaching levels more than 5 times higher than the maximum permitted level. These exceedances suggest that regular consumption of such eels may pose a health risk, particularly due to the bioaccumulative and toxic nature of dioxins and PCBs. Chronic exposure to these compounds has been associated with immunotoxicity, endocrine disruption, and increased cancer risk [7]. Based on the EFSA Tolerable Weekly Intake (TWI) for dioxins and DL-PCBs of 2 pg WHO-TEQ/kg body weight, even occasional consumption of highly contaminated eels could lead to exposure levels surpassing the recommended safety threshold, especially in vulnerable populations such as children and pregnant women.

## 5. Conclusions

This study provides evidence of substantial contamination of eels from Lake Garda with dioxin-like (TEQ Diox+PCB-DL) and non-dioxin-like PCBs (PCB-NDL), with a notable proportion of samples exceeding the maximum levels set by European Union regulations. Statistical analyses revealed significant correlations between contaminant concentrations and biometric parameters, particularly eel length and lipid content. TEQ Diox+PCB-DL and PCB-NDL concentrations were inversely related to body length and positively correlated with lipid percentage, reflecting the bioaccumulative behavior of these lipophilic compounds.

Despite these correlations, the multivariable models had limited explanatory power (adjusted R^2^ = 0.23 for TEQ Diox+PCB-DL; 0.17 for PCB-NDL), suggesting that additional unmeasured factors influence contaminant accumulation. Multinomial logistic regression further supported the role of length and lipid content in predicting contamination categories, although classification performance was limited (AUC = 0.63), likely due to group overlap among groups and unaccounted-for variability.

These findings raise concerns about the safety of consumption of eels from this region and support the continuation of the ban on eel fishing in Lake Garda. Future research should consider integrating broader environmental and ecological variables to improve predictive accuracy and deepen understanding of contaminant dynamics in aquatic ecosystems.

## Figures and Tables

**Table 1:** Multivariable linear model output for TEQ Diox+PCB-DL and PCB-NDL compounds.

	Variable	Coefficient	Standard Error	*p*-Value
TEQ Diox+PCB-DL (pg/g)	Intercept	7.625	7.174	0.291
	Length (cm)	−0.214	0.079	0.008
	Lipid (%)	0.756	0.160	<0.001
PCB-NDL (ng/g)	Intercept	229.85	96.031	0.019
	Length (cm)	−3.854	1.038	<0.001
	Lipid (%)	6.382	2.113	0.003

**Table 2:** Number of eels per classification category for each compound.

TEQ Diox+PCB-DL		
Irregular	Regular	Regular/uncertain
28	46	16
PCB-NDL		
Irregular	Regular	Regular/uncertain
5	84	1
Overall		
Irregular	Regular	Regular/uncertain
28	46	16

**Table 3:** Multinomial logistic model output for TEQ Diox+PCB-DL classification.

	Variable	Coefficient	Standard Error	*p*-Value	Exp (Coefficient)
Irregular	Intercept	−1.088	2.222	0.625	0.337
	Length (cm)	−0.0658	0.026	0.012	0.936
	Lipid (%)	0.1932	0.053	<0.001	1.21
Regular/ Uncertain	Intercept	−3.134	2.378	0.187	0.043
	Length (cm)	0.0078	0.027	0.775	1.01
	Lipid (%)	0.0522	0.0589	0.376	1.05

## Data Availability

Data are contained within the article or Supplementary Material.

## Supplementary Materials

The following supporting information can be downloaded at https://www.mdpi.com/article/10.3390/toxics13080690/s1, Table S1: Descriptive information about eel samples (weight, length, fat percentage, contaminant concentrations); Table S2: Method limit of quantification; Table S3: Descriptive statistics of TEQ Diox+PCB-DL and PCB-NDL compounds; Table S4: Descriptive statistics for each physical variables according to the classification of Diox+PCB-DL, PCB-NDL and “overall”.
