# Supplementary material for: Monitoring of PCDD/Fs and PCBs in European Eels (Anguilla anguilla) from Lake Garda: A Persistent Environmental Concern
Source: Toxics. 2025 Aug 19;13(8):690. doi: 10.3390/toxics13080690 (PMC12389985; doi:10.3390/toxics13080690)
Supplement: Supplementary file 1 [file toxics-13-00690-s001.zip › Supplementary Tables S2-S4.pdf]

Article

Monitoring of PCDD/Fs and PCBs in European Eels (*Anguilla anguilla*) from Lake Garda: A Persistent Environmental Concern

Federica Gallocchio [1,*], Marzia Mancin [1,*], Aurora Boscolo Anzoletti [1], Roberto Angeletti [1], Giancarlo Biancotto [1], Giorgio Fedrizzi [2], Mara Gasparini [2], Barbara Angelone [2], Silvana Bontacchio [2], Sabrina Di Millo [2], Francesca Cito [3], Gianfranco Diletti [3] and Giuseppe Arcangeli [1]

[1] Istituto Zooprofilattico delle Venezie, Viale dell'Università, 10, 35020 Legnaro, Italy; aboscolo@izsvenezie.it (A.B.A.); rangeletti@hotmail.it (R.A.); gbiancotto@izsvenezie.it (G.B.); garcangeli@izsvenezie.it (G.A.)
[2] Istituto Zooprofilattico Sperimentale della Lombardia e Dell'Emilia Romagna, Via Antonio Bianchi, 7/9, 25124 Brescia, Italy; giorgio.fedrizzi@izsler.it (G.F.); mara.gasparini@izsler.it (M.G.); barbara.angelone@izsler.it (B.A.); sabrina.dimillo@izsler.it (S.D.M.)
[3] Istituto Zooprofilattico Sperimentale dell'Abruzzo e del Molise Giuseppe Caporale, Via Campo Boario, 1, 64100 Teramo, Italy; f.cito@izs.it (F.C.); g.diletti@izs.it (G.D.)
* Correspondence: fgallocchio@izsvenezie.it (F.G.); mmancin@izsvenezie.it (M.M.)

Supplementary Materials

Table S1. Descriptive information about eels' sample (weight, length, fat percentage, contaminants concentration) (see attached Excel file).

Table S2. Method limit of quantification

Compound	LOQ pg/g wet weight
2,3,7,8-TCDF	0,08
1,2,3,7,8-PeCDF	0,08
2,3,4,7,8-PeCDF	0,08
1,2,3,4,7,8-HxCDF	0,2
1,2,3,6,7,8-HxCDF	0,2
2,3,4,6,7,8-HxCDF	0,2
1,2,3,7,8,9-HxCDF	0,2
1,2,3,4,6,7,8-HpCDF	0,2
1,2,3,4,7,8,9-HpCDF	0,2
1,2,3,4,6,7,8,9-OCDF	0,4
2,3,7,8-TCDD	0,08
1,2,3,7,8-PeCDD	0,08
1,2,3,4,7,8-HxCDD	0,2
1,2,3,6,7,8-HxCDD	0,2

1,2,3,7,8,9-HxCDD	0,2
1,2,3,4,6,7,8-HpCDD	0,2
1,2,3,4,6,7,8,9-OCDD	0,4
PCB 81	10
PCB 77	10
PCB 123	10
PCB 118	100
PCB 114	10
PCB 105	100
PCB 126	1
PCB 167	10
PCB 156	100
PCB 157	10
PCB 169	1
PCB 189	10
	LOQ ng/g wet weight
PCB 28	2
PCB 52	2
PCB 101	2
PCB 153	2
PCB 138	2
PCB 180	2

Table S3. Descriptive statistics of TEQ Diox+PCB-DL and PCB-NDL compounds

Compound		Min	1°quantile	Median	Mean	3°quantile	Max	Dev. std
TEQ Diox+ PCB-DL (pg/g)	50-65	4.60	7.95	10.40	18.46	24.40	77.10	16.86
	65-80	1.70	6.30	10.10	13.26	17.20	35.70	9.32
	>80	3.10	5.70	8.35	12.39	13.93	53.20	11.21
	Overall	1.70	6.63	9.90	14.53	17.10	77.10	12.73
PCB-NDL (ng/g)	50-65	31.00	48.50	82.00	174.20	251.00	665.00	178.50
	65-80	14.00	47.0	66.00	134.80	144.00	1620.00	272.59
	>80	15.00	33.25	57.50	85.80	96.75	444.00	84.59
	Overall	14.00	43.50	65.50	130.26	152.80	1620.00	199.04

Table S4. Descriptive statistics for each physical variables according to the classification of Diox+PCB-DL, PCB-NDL and "overall"

Compound	Variable	Class	Min	1° quantile	Median	Mean	3° quantile	Max	Dev. std
TEQ Diox+PCB-DL (pg/g)	Length (cm)	REG	60.00	66.25	80.00	79.33	88.00	100.00	11.05
		IRR	59.00	65.00	75.00	73.68	79.25	92.00	9.07
		REG/ UNC	65.00	71.00	77.50	81.12	90.00	110.00	13.79
	Weight (kg)	REG	0.55	0.74	1.04	1.10	1.41	1.96	0.38
		IRR	0.51	0.73	0.84	0.89	0.99	1.81	0.30
		REG/ UNC	0.53	0.76	0.86	1.11	1.28	2.69	0.57

	Lipid percentage (%)	REG	19.37	23.81	25.93	27.08	30.11	38.77	4.77
		IRR	21.98	28.09	30.66	31.82	35.81	39.22	4.89
		REG/ UNC	16.97	23.19	27.41	28.56	35.22	39.17	6.69
PCB-NDL (ng/g)	Length (cm)	REG	59.00	65.00	79.00	78.52	88.00	110.00	11.21
		IRR	61.00	64.00	65.00	69.80	75.00	84.00	9.52
		REG/ UNC	65.00	65.00	65.00	65.00	65.00	65.00	/
	Weight (kg)	REG	0.51	0.74	0.93	1.05	1.28	2.69	0.41
		IRR	0.61	0.75	0.81	0.82	0.86	1.07	0.17
		REG/ UNC	0.73	0.73	0.73	0.73	0.73	0.73	/
	Lipid percentage (%)	REG	16.97	24.64	27.98	28.72	32.27	39.22	5.52
		IRR	26.60	28.11	29.66	31.91	37.24	37.94	5.30
		REG/ UNC	21.98	21.98	21.98	21.98	21.98	21.98	/
Overall (pg/g)	Length (cm)	REG	60.00	66.25	80.00	79.33	88.00	100.00	11.05
		IRR	59.00	65.00	75.00	73.68	79.25	92.00	9.07
		REG/ UNC	65.00	71.00	77.50	81.12	90.00	110.00	13.79
	Weight (kg)	REG	0.55	0.74	1.04	1.10	1.41	1.96	0.38
		IRR	0.51	0.73	0.84	0.89	0.99	1.81	0.30
		REG/ UNC	0.53	0.76	0.86	1.11	1.28	2.69	0.57
	Lipid percentage (%)	REG	19.37	23.81	25.93	27.08	30.11	38.77	4.77
		IRR	21.98	28.09	30.66	31.82	35.81	39.22	4.89
		REG/ UNC	16.97	23.19	27.41	28.56	35.22	39.17	6.69
